# Effects of Aerobic-Resistance Training and Nutritional Intervention on Adiponectin, Interleukin-6, and hs-CRP Concentrations in Men with Abdominal Obesity—A Randomized Controlled Trial

**DOI:** 10.3390/ijms26199500

**Published:** 2025-09-28

**Authors:** Karol Makiel, Aneta Targosz, Piotr Kosowski, Agnieszka Suder

**Affiliations:** 1 Department of Anatomy, Faculty of Physical Rehabilitation, University of Physical Culture, 31-571 Cracow, Poland; 2 Department of Physiology, Faculty of Medicine, Jagiellonian University Medical College, 31-531 Cracow, Poland; 3 Department of Petroleum Engineering, AGH University, 30-059 Cracow, Poland

**Keywords:** aerobic-resistance exercise, abdominal obesity, inflammatory markers, fiber, insulin resistance, atherogenic index

## Abstract

The objective of this study was to assess the changes in adiponectin concentrations and inflammatory markers in men with abdominal obesity following physical exercise and exercise combined with dietary intervention. This study included 44 males with abdominal obesity (mean age 34.7 ± 5.5 years, waist circumference [WC] 110.3 ± 8.5, BMI 32.0 ± 3.9), who were randomly assigned to three groups: a control group without interventions (CG, *n* = 12), an experimental group engaging in aerobic-resistance exercise (EG, *n* = 16) and a group engaging in aerobic-resistance exercise combined with an ad libitum high-protein, low-glycemic index carbohydrate diet (EDG, *n* = 16). Body composition metrics: the body fat-, fat-free mass-, and abdominal fat-to body mass (BF/BM, FFM/BM, ABD/BM) indexes and the body adiposity index (BAI), along with biochemical blood analyses—adiponectin (ADIPO), interleukin-6 (IL-6), high-sensitivity C-reactive protein (hs-CRP), Castelli-II Index (CRI II) and fasting glucose–insulin (FG/I) ratio—were measured at baseline and after the intervention. The effects of the interventions on the analyzed variables across groups were assessed using mixed ANOVA tests with post hoc comparisons. Effect size (ES) was also calculated using partial eta squared (*η*p^2^). The exercise intervention (EG) resulted in a significant reduction in the BAI (*p* < 0.01), insulin resistance FG/I (*p* < 0.02), and IL-6 concentrations (*p* < 0.01) and initiated an increase in ADIPO secretion (*p* = 0.03). The combined intervention (EDG) reduced the insulin resistance FG/I (*p* = 0.02) and atherogenic index CRI II (*p* = 0.01), decreased inflammatory markers IL-6 (*p* = 0.01) by 48% and hs-CRP (*p* = 0.04) by 30%, and simultaneously increased the ADIPO (*p* = 0.02) concentration by 15%. These effects were accompanied by significant changes in body composition: reductions in visceral fat ABD/BM (*p* < 0.01), total fat BF/BM (*p* < 0.01), and BAI (*p* = 0.02) and an increase in FFM/BM (*p* < 0.01). A crucial role in achieving these outcomes was played by dietary modifications, i.e., the inclusion of low-glycemic index carbohydrates (*p* < 0.01), a 23% increase in protein intake (*p* < 0.01), and a 50% increase in dietary fiber intake (*p* < 0.01), which consistently deepened the energy deficit (*p* < 0.01) and reduced fat intake (*p* < 0.01). These findings underscore that short-term interventions, whether exercise alone or combined with dietary modifications, can effectively reduce inflammation and lower insulin resistance in men with visceral obesity. However, the combined intervention, involving both exercise and dietary modifications, resulted in more pronounced beneficial changes in both body composition and concentrations of adipokines, inflammatory markers, and atherogenic indices and insulin resistance.

## 1. Introduction

The primary tools in the treatment of obesity are the introduction of physical activity [1] and the implementation of an appropriate diet [2]. Individuals with excess adipose tissue should engage in 225 to 420 min of moderate-intensity physical activity per week to achieve clinically significant weight loss [3], or more than 150 min of vigorous aerobic activity, or an equivalent combination of moderate and vigorous intensity throughout the week, which provides additional health benefits (provided there are no contraindications due to comorbid conditions) [4]. According to current recommendations, engaging in moderate physical activity for 30–60 min daily or participating in vigorous exercise three times a week for 50 min necessitates the implementation of a specialized diet that treats the obese individual as an “athlete in training” [5,6,7]. Such an approach may also foster a positive self-image in patients with obesity, enabling them to view themselves as physically active individuals, thereby mitigating the psychological consequences associated with the societal stigmatization of obesity [8,9].

The formulation of a specialized dietary strategy for individuals with excess body weight who participate in physical activity should consider the specific type of exercise undertaken, its intensity and overall volume, and the presence of obesity-related comorbidities and the potential risk of developing metabolic syndrome [10,11]. Combining aerobic and resistance training may yield greater benefits than aerobic exercise alone, particularly in terms of reducing body fat levels, pro-inflammatory adipokines (such as leptin and IL-6), asprosin, and insulin resistance markers after 12 weeks of intervention [12,13,14]. The inclusion of resistance training in obese patients aims not only to increase energy expenditure but also to preserve or enhance fat-free mass during the long-term fat reduction process. This form of exercise primarily activates type II muscle fibers, whose recruitment during resistance workouts triggers the Akt–mTOR–p70S6K signaling pathway, consequently increasing the body’s demand for amino acids [15,16,17,18]. As a result of these physiological processes, dietary protein intake should be increased, particularly during weight reduction phases [19]. Higher protein intake recommendations also apply to individuals with obesity, mainly due to protein’s high thermic effect, prolonged satiety induced by amino acid availability, and beneficial effects on parameters associated with metabolic syndrome [20]. In the nutrition of physically active individuals, including those with obesity, the consumption of high-quality protein sources is recommended, especially those rich in branched-chain amino acids (BCAAs) [15,20]. An increase in dietary protein content should be accompanied by a higher intake of alkalizing compounds, with vegetables and fruits being the primary sources [21]. To ensure effective ATP regeneration under anaerobic conditions and the efficient replenishment of glycogen—particularly in type II muscle fibers, which are predominantly engaged during resistance training—adequate carbohydrate intake must be considered in the diet [22]. In elite athletes, the inclusion of simple sugars, disaccharides, and starches (especially amylopectin) is recommended [23], that is, carbohydrates with a high glycemic index. In individuals with obesity, carbohydrates should also serve as the main energy source; however, they should be characterized by a low glycemic index and high dietary fiber content [24]. Qualitative modification of carbohydrate intake toward those with a low glycemic index leads to reduced postprandial glycemia, decreased insulin resistance, fat mass reduction, and a lower risk of cardiovascular events. Lowering the glycemic index of the diet also contributes to prolonged satiety, thereby increasing the likelihood of sustained weight loss in individuals with obesity [25]. Given the benefits of a low-GI diet in obese individuals, it is advisable to include foods rich in amylose, polyphenols, and dietary fiber, and to combine protein and fat with starchy foods within meals to further attenuate postprandial glycemic responses [26].

The introduction of dietary intervention and physical training in patients with obesity leads to alterations in the secretion of adipose tissue-derived hormones, including adiponectin and interleukin-6 (IL-6) [12,27,28]. Interleukin-6 (IL-6) is a cytokine that exhibits both pro-inflammatory and anti-inflammatory properties. During physical exertion, skeletal muscles increase IL-6 secretion, which enhances lipolysis, improves insulin sensitivity in muscle tissue, and temporarily suppresses immune system function. However, the rise in IL-6 concentration in response to exercise is transient in nature [29]. In contrast, chronic low-grade IL-6 secretion is observed in necrotic adipose tissue, where pro-inflammatory M1 macrophages infiltrate and produce IL-6, thereby indicating a state of inflammation within the adipose tissue [30].

Adiponectin, which possesses anti-hyperglycemic, anti-atherogenic, and anti-inflammatory properties, may have significant clinical relevance in the development of therapies aimed at preventing obesity and its associated diseases [31]. Reduced adiponectin secretion has been documented in obesity and other metabolic disorders, whereas weight loss has been shown to increase adiponectin concentrations [32]. An inverse correlation has been demonstrated between adiponectin levels and BMI, total fat mass, as well as the intensity of systemic inflammation, as measured by hs-CRP and other inflammatory markers [33].

The aim of this study is to evaluate the effects of combined resistance and aerobic training with a high-protein diet containing low-glycemic index carbohydrates on serum concentrations of adiponectin, interleukin-6, and C-reactive protein (CRP) in men with abdominal obesity, as well as to compare the outcomes with those observed in a group performing resistance-aerobic training alone and in a control group. Males with abdominal obesity represent a high-risk target group in which testing the combined effects of concurrent aerobic–resistance training and a high-protein, low-GI diet is of particular clinical relevance. Restricting the sample to one sex also reduces hormonal heterogeneity, enabling a clearer assessment of the intervention’s effects on adipokine profiles and inflammatory markers. It was hypothesized that the combination of aerobic-resistance training with a high-protein, low-glycemic index carbohydrate diet would lead to more beneficial alterations in adipokine profiles, greater reductions in inflammatory markers, and more significant improvements in body composition than aerobic-resistance training alone in men with abdominal obesity.

## 2. Results

In accordance with the objectives of the project, the implementation of training interventions led to a significant increase in energy expenditure associated with structured exercise activity among participants in the EG (participated in a combined aerobic-resistance exercises) (*p* < 0.01) and EDG (underwent both aerobic-resistance training and followed a high-protein, low-glycemic index carbohydrate diet) (*p* < 0.01) groups, while no change was observed in the CG group (Table 1). The magnitude of the time effect (*η*p^2^ = 0.95) indicates a very strong modification in physical activity levels. The level of spontaneous physical activity (non-exercise activity thermogenesis) remained unchanged in all groups throughout the duration of the project (Table 1). Before the intervention, no significant differences were found between the groups in the analyzed parameters.

In the EDG group a significant decrease in the body fat-to-body mass ratio (BF/BM) was observed over time (*p* < 0.01), as well as in the intervention-by-time interaction (*p* < 0.01) (Table 2).

Similarly, the ABD/BM ratio also showed a significant reduction in the EDG group (*p* < 0.01). A significant change in the fat-free mass-to-body mass ratio (FFM/BM) was observed over time (*p* = 0.04) and in the interaction effect (*p* < 0.01); the proportion of fat-free mass relative to total body mass increased in the EDG group (*p* < 0.01). The body adiposity index (BAI) demonstrated a significant change over time (*p* = 0.02), and a significant intervention-by-time interaction was confirmed (*p* < 0.01). BAI decreased significantly in both the EG (*p* < 0.01) and EDG (*p* < 0.01) groups (Table 2).

Before the intervention, no significant differences were found between the groups in the analyzed parameters.

**Figure 1:**
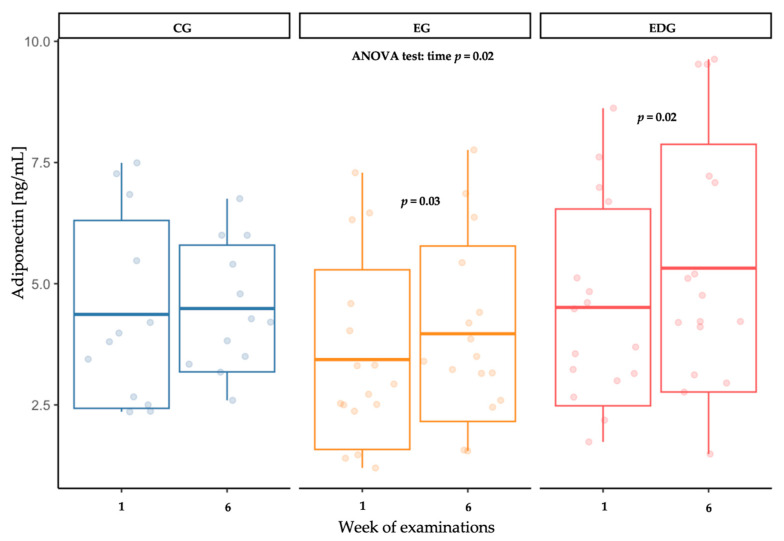
Changes in adiponectin (ADIPO) concentration [ng/mL] in the control (CG), exercise (EG) and exercise-diet (EDG) groups at baseline and after six weeks.

The two-way mixed-design analysis of variance (ANOVA) indicated a significant time effect on adiponectin (ADIPO) concentration (*p* = 0.03). The change in ADIPO concentration after 6 weeks was confirmed in both EG (*p* = 0.03) and EDG (*p* = 0.02) (Table 3, Figure 1).

**Figure 2:**
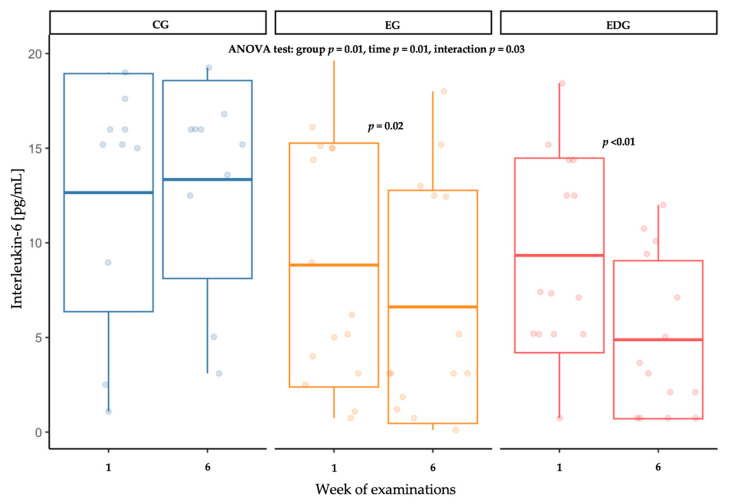
Changes in interleukin-6 (IL-6) concentration [pg/mL] in the control (CG), exercise (EG) and exercise-diet (EDG) groups at baseline and after six weeks.

Significant differences were confirmed in interleukin-6 (IL-6) concentrations between group (*p* = 0.01), in time (*p* = 0.01) and in the interaction of time and intervention type (*p* = 0.03). Post hoc tests confirmed decrease in IL-6 in intervention group: in EG IL-6 was decrease by 30% (*p* = 0.02) and in EDG group by 48% (*p* < 0.01). In the control group (CG), no significant change in IL-6 concentration was observed over the 6-week period. Furthermore, post-intervention IL-6 levels were significantly higher in the CG compared to both the EG (*p* = 0.01) and EDG (*p* = 0.01) groups (Table 3, Figure 2).

The CRP concentration decrease over time in EDG group (*p* = 0.04) (Table 3). Castellie Index (CRI II) decreased over the six weeks (*p* = 0.01) in EDG group. A significant change over time was also observed for fasting glucose–insulin (FG/I) ratio (*p* = 0.02) in both intervention groups, EG (*p* = 0.02) and EDG (*p* = 0.02) (Table 3).

Before the intervention, no significant differences were found between the groups in the analyzed parameters.

A change in dietary energy intake over time (*p* = 0.02) as well as the interaction between group and time (*p* < 0.01) was indicated. (Table 4). Post hoc tests confirmed a decrease in the level of calories in the EGD group (*p* < 0.01).

Significant changes occurred in proteins intake considering the interaction of time and type of intervention (*p* < 0.01). This variability was also confirmed by a high effect size (*ƞ*p^2^ = 0.13) for the interaction. Post hoc tests also indicated an increase in protein intake at 0.34 g/kg-bm in the EGD group after 6 weeks of intervention (*p* < 0.01) (Table 4).

Carbohydrates intake also changed significantly over time (*p* = 0.03), and the interaction between time and group was also significant (*p* < 0.01) (Table 4). Participants in the EGD group significantly decrease carbohydrates intake during the time (*p* < 0.01) and interaction (*p* < 0.01) after 6 weeks.

Dietary fiber levels also changed, considering the interaction of time and group (*p* < 0.01) with a moderate effect size (*ƞ*p^2^ = 0.08). Participants in the EGD group increase dietary fiber intake by 50% (*p* < 0.01) (Table 4).

The fats intake changed in the interaction between the type of intervention and time (*p* < 0.01). The changes also confirmed the high effect size (*ƞ*p^2^ = 0.10). The reduction in fats level in the EGD group was 26% after 6 weeks (*p* < 0.01) (Table 4).

Before the intervention, no significant differences were found between the groups in the analyzed parameters.

The applied multiple regression model demonstrated that both FG/I and CRI II were significantly connected with the concentration of ADIPO (*p* < 0.01). The analyzed variables explained 39% of variability in ADIPO (value of R^2^ model = 0.39) (Table 5).

The applied multiple regression model demonstrated that FAT/BM was significantly connected with the concentration of hs-CRP (*p* < 0.01). The analyzed variables explained 15% of variability in hs-CRP (value of R^2^ model = 0.15) (Table 6).

## 3. Discussion

The implementation of the training intervention alone was associated with improvements in adipokine concentrations and the insulin resistance index. However, the combined intervention involving both training and dietary modification resulted in more pronounced beneficial changes in the concentrations of the evaluated adipokines, the insulin resistance index, atherogenic indices, and hs-CRP levels. Regarding body composition, a reduction in body fat relative to total body mass was confirmed, along with a decrease in the ABD/BM index, indicating a substantial reduction in visceral adipose tissue. Concurrently, an increase in the fat-free mass to body mass ratio (FFM/BM) was observed, reflecting favorable changes in body composition in the group undergoing the combined training and dietary intervention. No significant changes in body composition were observed in the other study groups. In the intervention groups, a significant reduction in the body adiposity index (BAI) was noted. In the dietary domain, a significant decrease in caloric intake—primarily from carbohydrates—was confirmed in the EDG group, along with an increase in dietary protein and fiber intake. These dietary adjustments contributed to the more favorable outcomes of the intervention.

Increasing dietary protein intake in individuals with obesity may raise concerns among researchers due to potential risks associated with metabolic and clinical complications, such as bone mass loss and renal dysfunction. Obesity is recognized as a significant risk factor for the development of chronic kidney disease (CKD). It may contribute to kidney damage through mechanisms such as glomerular hyperfiltration, oxidative stress, and chronic inflammation. Weight reduction in individuals with obesity is recommended as a strategy to lower the risk of CKD progression. Moreover, epidemiological studies indicate that individuals with obesity have a two- to threefold higher risk of developing CKD compared to those with normal body weight. Weight loss has been shown to improve renal function and reduce disease progression [34]. Despite reports suggesting that a high-protein diet may be harmful to individuals with pre-existing kidney dysfunction, there is limited evidence indicating that high protein intake poses a health risk to individuals with normal renal function [35]. The primary argument commonly raised in discussions concerning the risks associated with increased dietary protein intake is the well-established clinical recommendation to restrict protein consumption in patients with chronic kidney disease, typically to a level of 0.6 g/kg of body weight, in order to slow disease progression [36,37]. Based on this, it is often hypothesized that a high-protein diet contributes to the deterioration of kidney function. However, a review of studies in this area does not demonstrate a link between increased dietary protein intake and the development of kidney or bone diseases [37]. A high-protein diet is typically defined as protein intake exceeding 1.5 g/kg per day.

Athletes, particularly those engaged in physique-focused disciplines, often adopt high-protein diets to optimize muscle protein balance, increase fat-free mass, and enhance performance. In studies involving individuals participating in such activities and performing resistance training, no increased risk of kidney disease was observed [38]. Nevertheless, there is strong evidence indicating a risk of kidney damage associated with the use of anabolic-androgenic steroids, excessive intake of vitamins A, D, and E, dehydration practices, abuse of diuretics, and the use of nonsteroidal anti-inflammatory drugs (NSAIDs) [39]. Physique athletes often exceed the recommended protein intake levels, with dietary protein contributing up to 44% of total energy intake, equating to 3.71 g/kg of body weight [40]. Considering the potential risks associated with excessive protein consumption in individuals with obesity, we increased dietary protein intake to 1.5 g/kg of body weight, representing 25% of total energy intake. This approach aligns with established nutritional guidelines for physically active individuals with obesity and complies with the recommendations of the International Olympic Committee on athlete nutrition [24,41]. It is important to note that participants in the intervention groups engaged in intensive physical activity three times per week. Increasing protein intake in individuals who do not engage in structured training does not yield additional benefits in terms of muscle mass or strength development, aerobic performance [42] or, most likely, overall health improvements.

A key component of the intervention was the combination of increased dietary protein with a low-glycemic-index diet. This was achieved through the inclusion of a high intake of vegetables, fruits, and whole grain products, thereby meeting the recommendations for the alkalizing potential of a plant-based diet and enhancing the intake of plant-derived protein, which is considered more beneficial to overall health, including kidney function [21]. Significant benefits of the applied dietary intervention combined with training included favorable changes in body composition. Nutritional recommendations for athletes aim, among other goals, to maintain or promote muscle mass and to support glycogen replenishment following physical activity. To achieve optimal results in this regard, it is recommended to consume a combination of carbohydrates and protein—particularly in the post-exercise meal—within two hours after exercise [43] during the so-called “anabolic window” [44]. In our project, participants most commonly consumed, after training, whey protein or a high-protein yogurt combined with oats and a low-glycemic-index fruit, such as berries, pear, apple, or peach. Such a combination has been shown to significantly enhance both muscle glycogen synthesis and muscle protein synthesis. Post-exercise consumption of glucose together with whey protein hydrolysate (WPH) markedly increases the levels of phosphorylated Akt/PKB (by 131%) and phosphorylated PKCζ (by 154%) compared to glucose intake alone following exercise [45]. Moreover, post-exercise intake of whey protein in combination with amylopectin and chromium stimulates muscle protein synthesis by increasing mTOR levels by 224.2% [46]. In our study, we achieved an increase in fat-free mass despite overall weight loss, through the implementation of a well-designed combination of dietary intervention and physical training. Weight reduction strategies based on suboptimal or unfavorable interventions often result primarily in the loss of muscle mass [47]. In contrast, in our study, the observed reduction in body fat, particularly in visceral fat stores, represents a key therapeutic target in obesity treatment.

The application of training alone did not lead to a significant reduction in visceral adipose tissue. Ross et al. [48] reported different findings, where physical activity alone resulted in visceral fat reduction even without significant body weight loss. In our study, training without dietary intervention did not produce such results, emphasizing the substantial benefits of combining multiple strategies that simultaneously stimulate fat-free mass gain and fat mass reduction in the treatment of obesity.

Current sports nutrition guidelines recommend the consumption of low glycemic index (GI) carbohydrates prior to training [49] and high GI carbohydrates post-exercise to enhance glycogen resynthesis [50]. During our intervention, however, we chose to apply low-GI carbohydrates even in the post-exercise meal. In accordance with dietary recommendations, participants consumed carbohydrate-rich foods, but the grain products selected were high in amylose and dietary fiber, and low in amylopectin to maintain a low glycemic index [51], As a result, despite a relatively high carbohydrate intake, the insulin resistance index (FG/I) improved.

In the intervention groups, a significant reduction in inflammatory status was observed. An increase in adiponectin concentration was confirmed, by 15% in the group combining diet and training, and by 14% in the group undertaking resistance-aerobic training alone. A decrease in interleukin-6 (IL-6) concentration was also confirmed, with a reduction of 48% in the EDG group and 30% in the EG group. Furthermore, in the group combining diet and exercise, C-reactive protein (CRP) levels were reduced by 30%. These favorable changes underscore the value of engaging in physical training, even without dietary intervention, for improving health outcomes in individuals with obesity.

However, the initiation of physical activity does not always result in significant changes in adiponectin (ADIPO) concentrations. Both acute and chronic exercise may be insufficient to induce a clinically relevant decrease in this adipokine [52,53]. Achieving meaningful changes in body composition increases the likelihood of raising ADIPO levels in individuals with obesity [54,55], thus supporting the recommendation to include dietary intervention to optimize health outcomes [56]. Adiponectin (ADIPO) concentrations and their variability in response to the applied interventions were closely associated with the monitored indices of insulin resistance (FG/I) and atherogenicity (CRI II). Multiple regression analysis revealed that an increase in ADIPO concentrations was accompanied by a reduction in insulin resistance and a decrease in the CRI II index, indicating a potential preventive effect of the interventions in the context of cardiovascular diseases. The confirmed reduction in insulin resistance in the intervention groups, along with the increase in ADIPO concentrations, suggests that both the combination of aerobic and resistance training and its integration with dietary modification lead to beneficial changes—most likely mediated by enhanced secretion of this adipokine. A similar association was confirmed in our previous studies [53]. Adiponectin plays a key role in metabolic homeostasis—it enhances tissue insulin sensitivity, promotes β-oxidation of fatty acids in skeletal muscle and liver, and exerts anti-inflammatory effects by reducing the production of pro-inflammatory cytokines [57]. Hypoadiponectinemia is associated with impaired glucose regulation, chronic low-grade inflammation, obesity, atherosclerosis, and type 2 diabetes [58]. In abdominal obesity, elevated levels of IL-6 and hsCRP are likely indicative of an underlying inflammatory state. Our multiple regression model confirmed the association between a persistent low-grade inflammatory state, as reflected by hs-CRP levels, and increased adipose tissue content among study participants. Both IL-6 and its associated biomarker CRP are important predictors of type 2 diabetes and all-cause mortality. Moreover, genetically reduced IL-6 expression has been linked to a more favorable cardiometabolic profile [59,60] observed in our study should be interpreted as beneficial improvements in overall health status. Inflammatory markers showed substantial between-subject dispersion—e.g., CRP–IL-6 in EG (M = 6.17; SD = 6.17; CV ≈ 100%) and hs-CRP in EDG (M = 2.69; SD = 2.34; CV ≈ 87%)—consistent with right-skewed distributions and biological heterogeneity. Nevertheless, significant time and group × time effects persisted in the multifactor repeated-measures ANOVA after log transformation of relevant biomarkers and in nonparametric sensitivity analyses, indicating a coherent intervention effect beyond random scatter. The alterations in adipokine concentrations observed in both intervention groups further emphasize the positive impact of physical activity on health, a finding supported by other researchers as well [61,62].

### Limitations

Despite the significant changes observed in inflammatory markers, including reductions in CRP and IL-6 concentrations, as well as an increase in adiponectin levels, the interpretation of the underlying mechanisms remains incomplete due to three key methodological limitations.

In the present study, no targeted nutritional intervention was implemented to maintain an anti-inflammatory fatty acid profile. The intervention primarily focused on the intake of high-quality protein and low-glycemic index carbohydrates, whereas the quantity and composition of dietary lipids were indirectly shaped by changes in the consumption of other macronutrients.

The implementation of a diet that systematically monitors the composition of the lipid fraction—specifically aiming to achieve an approximately 1:1 ratio of omega-3 to omega-6 polyunsaturated fatty acids, along with a reduction in the intake of pro-inflammatory saturated and trans fatty acids—could potentially yield more favorable outcomes in terms of inflammatory biomarkers such as CRP, IL-6, and adiponectin [63].

Another limitation of the present study is the lack of parallel monitoring of gut microbiome composition. An increasing body of evidence suggests that lifestyle modifications involving diet and physical activity can significantly influence the microbial ecology of the gastrointestinal tract, which in turn feeds back into host metabolism through the production of short-chain fatty acids, regulation of the gut–liver axis, and modulation of inflammatory responses. The absence of metagenomic analyses (e.g., 16S rRNA sequencing or shotgun metagenomics) limits the ability to elucidate the mechanisms underlying the observed metabolic and inflammatory changes. Incorporating omics-based tools into future research protocols would enable a more integrated interpretation of the relationships between diet-and-exercise interventions, metabolic adaptations, and the potential role of the gut microbiota [64,65].

Another relevant area of omics research is epigenomics, and one of the key epigenetic mechanisms is DNA methylation. DNA methylation regulates gene expression without altering the DNA sequence, and its analysis enables the investigation of these regulatory changes as well as the estimation of biological age. In the context of interventions combining aerobic-resistance training and nutritional, combining DNA methylation analysis and epigenetic age assessment could provide a more comprehensive understanding of the biological changes occurring in study participants.

DNA methylation-based biomarkers offer the ability to estimate biological age, which is increasingly recognized as a sensitive indicator of cumulative lifestyle and environmental exposures. Observational studies have demonstrated associations between DNA methylation patterns and various lifestyle factors. For example, regular physical activity has been linked to slower biological aging, while diets rich in vegetables are associated with lower epigenetic age acceleration, and meat-rich diets with accelerated aging [66].

This study enrolled men only, which limits generalizability. We deliberately focused on men because adult males experience an earlier onset and a higher burden of cardiovascular mortality than age-matched females [67], which is linked, in part, to greater visceral (abdominal) fat accumulation and unfavorable adipokine profiles [68]. Men more commonly exhibit central adiposity, predisposing to insulin resistance, dyslipidemia, and hypertension. They also tend to have lower adiponectin and higher pro-inflammatory mediators (e.g., IL-6 [69]), reflecting low-grade inflammation and elevated cardiometabolic risk. Consequently, men with abdominal obesity represent a high-risk target group in which testing the combined effects of concurrent aerobic-resistance training and a high-protein, low-GI diet is of particular clinical relevance. Restricting the sample to one sex also reduces hormonal heterogeneity, enabling a clearer assessment of the intervention’s effects on adipokine profiles and inflammatory markers.

Well-described sex differences in fat distribution (greater peripheral/gynecoid fat in premenopausal women), adipokine profiles (e.g., higher circulating adiponectin in women [70]), and the endocrine milieu may lead to different magnitudes or trajectories of response to concurrent training and high-protein, low-GI diets. We therefore cannot assume equivalence in females, and future trials should include women—across hormonal stages—and participants with obesity beyond the abdominal phenotype.

Additionally, the intervention duration precludes inference about long-term clinical outcomes (incident cardiovascular disease or type 2 diabetes). The observed changes in adipokines and inflammatory markers constitute surrogate endpoints; whether similar or greater benefits persist and translate into fewer clinical events will require longer follow-up, sustained adherence, and adequately powered outcome studies. Finally, the narrow inclusion criteria enhance internal validity but constrain external validity, and replication in broader, more diverse populations is warranted.

Another limitation of the study is the lack of mechanistic assays; biopsies and molecular analyses of anabolic/catabolic signaling (e.g., phosphorylation of Akt, mTOR, and p70S6K), mitochondrial function, intramuscular glycogen, or muscle fiber characteristics. Consequently, our findings on adipokines, inflammatory markers, and body composition cannot be causally linked to specific tissue-level pathways. Future trials should incorporate mechanistic endpoints—including muscle and, where feasible, adipose tissue biopsies—together with targeted proteomics/metabolomics to delineate how concurrent training and a high-protein, low-GI diet modulate skeletal muscle and adipose signaling.

## 4. Materials and Methods

### 4.1. Study Design

This study was designed as a prospective, randomized, controlled trial. The variables presented in this manuscript represent a subset of a broader research project investigating the effects of exercise and dietary intervention on body composition, as well as biochemical and hormonal markers, in men with abdominal obesity [14,71].

Eligibility criteria included male sex, abdominal obesity, age between 30 and 40 years, and provision of written informed consent for voluntary participation. Participants were also required to submit a medical certificate confirming the absence of contraindications to combined aerobic and resistance exercise. Individuals with medical conditions such as myocardial ischemia, decompensated heart failure, cardiac arrhythmias, severe pulmonary hypertension, symptomatic aortic stenosis, acute myocarditis, endocarditis or pericarditis, uncontrolled hypertension, aortic dissection, Marfan syndrome, uncontrolled diabetes, psychiatric disorders, or any condition limiting mobility were excluded. Participants were not allowed to engage in any structured physical activity programs outside the study; however, routine daily physical activity was permitted.

Exclusion criteria included failure to provide a medical certificate confirming suitability for aerobic-resistance training and insufficient attendance in the intervention sessions, with the minimum required attendance set at above 90%. The use of antibiotics, anti-obesity medications, so-called “fat-burning” supplements, and adherence to vegan diets constituted exclusion criteria. Supplement and medication use was monitored, and participants were advised not to modify their ongoing pharmacotherapy during the study.

A total of 53 participants were randomly assigned to one of three study groups using a concealed allocation method. This process involved selecting opaque envelopes, each containing a group-assigned number. Group allocation for the intervention arms was coordinated by a designated trainer. Recruitment and implementation of the intervention were conducted between February 2022 and July 2022, ensuring that the final sample size met the minimum statistical power requirements.

All volunteers (n = 53) were provided with written information detailing the study’s objectives, procedures, and overall design. Each participant gave written informed consent for the processing of personal data, voluntary participation, and use of the results for scientific purposes. Informed consent was obtained from all individuals prior to study initiation. Participants retained the right to withdraw from the study at any time without consequence.

**Figure 3:**
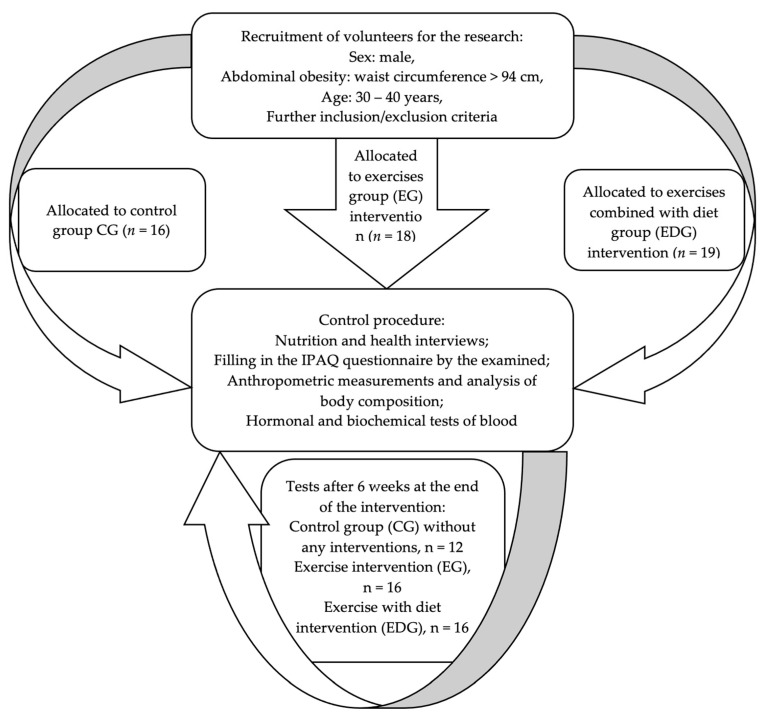
Study flowchart.

Participant withdrawals and exclusions were recorded and attributed to the following reasons: missing more than 10% of training sessions (2 cases), failure to adhere to dietary guidelines (3 cases), uncontrolled alcohol intake (1 case), and non-attendance at follow-up assessments (3 cases). During the course of the study, 4 participants from CG, 2 from EG, and 3 from EDG discontinued participation. Consequently, the final analysis included 44 participants. The participant flow is illustrated in Figure 3.

No adverse events or harms were reported during the study period. Due to the nature of the interventions and the absence of placebo or sham procedures, blinding was not implemented. However, to mitigate potential bias, the laboratory personnel, statistical analysts, and outcome assessors were blinded to group assignments.

This study received ethical approval from the Ethics Committee of the Regional Chamber of Physicians in Krakow (approval no. 15/KBL/OIL/2022) and was conducted in accordance with the Declaration of Helsinki. Additionally, the trial was registered in the Australian New Zealand Clinical Trials Registry under registration number ACTRN12624000184572 (26 February 2024), in compliance with CONSORT guidelines.

### 4.2. Sample

The study included 44 men aged between 30 and 40 years (mean age: 34.7 ± 5.5 years), all meeting the criteria for abdominal obesity, defined as a waist circumference greater than 94 cm. Participants were recruited via social media platforms from individuals employed within a large residential community in Kraków. They were randomly allocated into three groups:

Control group (CG): 12 men (mean age: 34.1 ± 5.5 years; body mass: 101.9 ± 11.5 kg; waist circumference: 111.2 ± 6.8 cm) who were informed about general obesity treatment recommendations but did not undergo any active intervention;

Experimental group—exercise (EG): 16 men (mean age: 34.8 ± 6.0 years; body mass: 105.6 ± 7.4 kg; waist circumference: 110.8 ± 11.3 cm) who participated in a combined aerobic and resistance exercise program;

Experimental group exercise–diet (EDG): 16 men (mean age: 34.9 ± 5.6 years; body mass: 105.6 ± 10.3 kg; waist circumference: 109.0 ± 7.6 cm) who underwent both aerobic-resistance training and followed a high-protein, low-glycemic index carbohydrate diet.

Baseline characteristics of the participants are presented in Table 7. No statistically significant differences were observed between the groups in terms of age or key anthropometric parameters at baseline.

### 4.3. Methods

Assessments were conducted for all participants at two points in time, before and after 6 weeks of the intervention:

#### 4.3.1. Anthropometric Measurements

Height (Ht, cm) was measured to the nearest millimeter using a stadiometer (Seca 231, Hamburg, Germany) with participants standing barefoot in an upright position, head oriented in the Frankfurt horizontal plane. Body mass (BM, kg) was determined using a calibrated medical scale (Beurer PS 240, Budapest, Hungary) with an accuracy of 50 g, with subjects standing in a relaxed posture. Waist circumference (WC, cm) was assessed using a non-elastic anthropometric tape, measured to the nearest millimeter at the midpoint between the lower margin of the rib cage and the upper border of the iliac crest, at the end of a normal exhalation. And hip circumference (cm) was measured at the level of the greatest protrusion of the buttocks, using a non-stretchable tape measure, with the subject standing upright and feet together. Body adiposity index (BAI) was calculated as [72]:

BAI = (Hip Circumference [cm]/Height [m]^1.5) − 18

#### 4.3.2. Body Composition

Body composition, including total body fat (BF, kg), abdominal fat mass (ABD, kg), and fat-free mass (FFM, kg), was assessed using dual-energy X-ray absorptiometry (DEXA) with a Lunar Prodigy Primo PR + 352163 scanner (GE Healthcare, Chicago, IL, USA).

#### 4.3.3. Adipokines

Fasting venous blood samples were drawn in the morning after a 24 h rest period from training. Before blood collection, study participants were instructed to observe a 12 h fast, refraining from eating and from drinking any beverages other than water. On the day of sampling, they were to arrive fasting, after drinking 1–2 glasses of water. It was recommended that medications be taken after the blood draw. Blood was collected by trained nursing personnel from the basilic, cephalic, or median cubital vein into Vacumed^®^ tubes (F.L. Medical, Torreglia, Italy). Samples were centrifuged at 1000× *g* for 15 min at 4 °C (MPW-351R, MPW Med. Instruments, Warsaw, Poland). Serum was then aliquoted and stored at −80 °C (BIO Memory 690L, Froilabo, Paris, France) until analysis. Concentrations of adiponectin (ADIPO, ng/mL) and interleukin-6 (IL-6, pg/mL) were determined using commercially available enzyme-linked immunosorbent assay (ELISA) kits, following the manufacturers’ instructions. The human adiponectin ELISA Kit (Cat. No. E09) was purchased from Mediagnost (Reutlingen, Germany), and IL-6 (Cat. No. E-3200) from LDN Labor Diagnostika Nord GmbH & Co.KG (Am Eichenhain, Nuremberg, Germany). Optical density readings at 450 nm were obtained with an ELx 808 microplate spectrophotometer (BioTek Instruments, Winooski, VT, USA). All analyses were conducted at the Laboratory of Genetics and Molecular Biology, Department of Physiology, Jagiellonian University Medical College, Kraków, Poland.

#### 4.3.4. Biochemical Blood Parameters

Plasma glucose (GLU, mg/dL) was quantified using an enzymatic colorimetric method on a Cobas c701/702 analyzer (Roche Diagnostics International Ltd., Mannheim, Germany). Serum insulin (INS, µIU/mL) levels were assessed using electrochemiluminescence immunoassay (ECLIA) on the Cobas e801 analyzer (Roche Diagnostics, Indianapolis, IN, USA), employing GLUC3 and Elecsys Insulin-specific reagents per the manufacturer’s protocol. Total cholesterol (TC, mmol/L) and high-density lipoprotein cholesterol (HDL-C, mmol/L) were analyzed using spectrophotometry on an Architect ci-4100 clinical chemistry analyzer (Abbott Laboratories, Wiesbaden, Germany). The intra-assay and inter-assay coefficients of variation (CVs) for all biochemical assessments ranged between 0.9 and 1.2% and 1.2–1.8%, respectively. The low-density lipoprotein cholesterol (LDL-C) concentration was calculated using the standard Friedewald equation [73]:

LDL-C (mmol/L) = TC (mmol/L) − HDL-C (mmol/L) − (TG (mmol/L)/2.2)

The Castelli Risk Index II (CRI II) was calculated using the following formula [74]:

CRI II = LDL-C (mmol/L)/HDL-C (mmol/L)

The fasting glucose–insulin (FG/I) ratio was calculated using the following formula [75]:

FG/I = Glucose (mg/dL)/Insulin (µIU/mL)

The analysis of high--sensitivity c-reactive protein (hs-CRP, mg/L) was performed using the immunoturbidimetric method on the Alinity C analyzer (Abbott, Abbott Park, IL, USA), employing Abbott reagents, version G84783R02, 09-2019.

#### 4.3.5. Assessment of Total Energy Expenditure and Dietary Energy Intake

To evaluate non-exercise activity thermogenesis (NEAT), the International Physical Activity Questionnaire (IPAQ) in its long form was administered across all three study groups. This self-reported tool was employed to estimate total daily energy expenditure derived from habitual physical activity [76,77]. In addition, energy expenditure specifically attributable to the training sessions in the intervention groups was measured using a Polar M200 GPS Running Watch equipped with a wrist-based heart rate monitor (Kempele, Finland).

Dietary energy intake was assessed via a 24 h dietary recall conducted by a certified clinical dietitian using the dietary record method. Three dietary recalls were obtained for each evaluation point (baseline and six weeks), including two weekdays and one weekend day. The mean intake across the three days was used to estimate total energy and nutrient consumption. Dietary data were processed using the DietaPro software (version 4.0, Institute of Food and Nutrition, Warsaw, Poland), allowing for a quantitative analysis of macronutrient composition and dietary modifications throughout the intervention period. The generated reports included intake of protein (%), carbohydrates (%), dietary fiber (g), and fat (%). These data were also used to calculate the fiber-to-energy ratio of the diet, expressed in megajoules (mJ).

In the intervention group receiving both exercise and dietary modification, qualitative dietary adjustments included an increase in protein intake and the incorporation of low glycemic index foods (see detailed protocol in Section 4.4.2). In addition to the 24 h recalls, participants in this group recorded daily food intake using the Fitatu dietary tracking application (version 3.41, Fitatu Ltd., Poznań, Poland), which facilitated enhanced adherence monitoring. Each training session was accompanied by a review of dietary entries by the clinical dietitian, who also provided individualized feedback and consultation to ensure compliance with the dietary intervention.

### 4.4. Interventions

#### 4.4.1. Aerobic Resistance Exercises

Participants assigned to groups EG and EDG engaged in a structured exercise intervention conducted over a six-week period at a local fitness facility, under the supervision of a certified personal trainer. Training sessions were held three times per week, each lasting 60 min, and consistently scheduled between 6:00 and 9:00 p.m. All sessions were led by the same instructor to ensure consistency in training delivery. The exercise environment was standardized, with the room maintained at a constant temperature of 22 °C and relative humidity of 45%. Compliance with the intervention protocol was monitored via an attendance log. Participants missing more than 10% of scheduled sessions were excluded from the final analysis.

Prior to initiating the main training protocol, a one-week preparatory phase was implemented, during which participants received practical instruction on proper technique for aerobic-resistance exercises. This period facilitated neuromuscular adaptation and ensured appropriate execution of the training regimen. Muscle strength was assessed using the one-repetition maximum (1 RM) test, a validated method for determining maximal voluntary strength in field-based settings.

The aerobic-resistance training program (see Supplementary Material Table S1) was conducted in small groups (maximum of five participants) to enhance individual supervision and safety. Each session began with a five-minute aerobic warm-up on a treadmill at 50% of the participant’s maximum heart rate (HR-max). The resistance component followed a “push–pull” model, alternating training of opposing muscle groups. “Pull” sessions targeted the gluteals, hamstrings, latissimus dorsi, posterior deltoids, and biceps, whereas “push” sessions focused on the quadriceps, pectorals, anterior/lateral deltoids, and triceps. Each resistance session comprised six exercises, performed in four sets of 12 repetitions at 70% of 1 RM, with 60 s rest intervals between sets.

Following resistance training, participants completed a 10 min aerobic segment at 70% HR_max, utilizing a treadmill (Technogym New Excite Run Now 500, Cesena, Italy), stationary bicycle (Technogym Artis, Cesena, Italy), or elliptical trainer (Precor EFX556i Elliptical, Woodinville, WA, USA). Equipment selection was rotated to minimize the risk of overuse injuries in the lower extremities. Sessions concluded with a five-minute cooldown, including diaphragmatic breathing and static stretching.

Previous analyses conducted within the framework of this research demonstrated that both the aerobic-resistance training alone (EG) and its combination with a dietary intervention (EDG) led to significant increases in total daily energy expenditure (kcal/day) after six weeks, compared to baseline values [14]. Specifically, post-intervention energy expenditure reached 2871.9 ± 286.1 kcal/day in EG and 2846.7 ± 263.9 kcal/day in EDG. Additionally, a significant increase in training load (sum of 1 RM from squat, bench press, and lat pulldown exercises) was observed, with EG showing a 4.5% increase and EDG a 7% increase after the intervention period.

#### 4.4.2. High-Protein, Low-Glycemic Index Carbohydrate Diet

The nutritional intervention for EDG was based on a high-protein dietary model incorporating carbohydrates with a low glycemic index (GI). Given the inherent difficulty in sustaining such a regimen—especially in maintaining appropriate caloric balance over a prolonged period—the core objective of the dietary education component was to emphasize specific food groups rather than rigid calorie control. The primary energy sources were carbohydrates characterized by a low GI, including non-starchy vegetables, selected fruits, and whole grain products. Protein intake was emphasized as the second key macronutrient, primarily sourced from lean animal products such as low-fat dairy, poultry, meat, and fish.

The dietary modifications were designed to induce a passive (unintentional) caloric deficit of approximately 500 kcal/day, with the aim of achieving a gradual weight reduction of about 0.5 kg per week—totaling an estimated loss of 3 kg over the six-week intervention period [78]. The intended macronutrient distribution was approximately 25% of total energy from protein, 50% from carbohydrates, and 25% from fats. Due to the high fiber content associated with low-GI carbohydrates, the expected daily fiber intake in EDG was assumed to exceed 30 g.

To address the potential variability in dietary adherence and ensure consistency in the application of dietary principles across participants, a comprehensive monitoring framework was implemented. Alongside routine dietary consultations, participants received individualized materials, including structured meal plans, shopping lists, and practical dietary guidelines. A modified “shopping center” strategy was adopted to simulate an ad libitum nutritional intervention, allowing participants to select food items freely within predefined constraints [79,80]. The higher satiety provided by selected food items facilitated the achievement of a sustained negative energy balance.

The nutritional intervention included the following structured components:

- Participants were instructed to shop exclusively in selected retail chains offering standardized products sourced from centralized warehouses to ensure consistency in food quality and availability.
- Visual aids were employed for educational purposes, including pictorial charts of high-protein foods and low-GI carbohydrate options. These materials were adapted from the Food and Nutrition Institute’s (Warsaw, Poland, 2000) photographic food atlas.
- A custom photo catalog featuring available food products from local shopping centers was provided to support appropriate food selection.
- Participants were equipped with precision kitchen scales (accuracy ± 1 g) to measure food portions prior to meal preparation or consumption.
- All food intake was logged using the Fitatu dietary application (version 3.41, Fitatu Ltd., Poznan, Poland), which generated real-time feedback on caloric and macronutrient consumption.
- A licensed dietitian continuously monitored dietary logs submitted via the app and provided individualized feedback to ensure compliance with dietary targets.
- When dietary deviations occurred—such as the inclusion of high-GI foods or insufficient protein intake—the dietitian recommended appropriate substitutions aligned with the study protocol.
- Participants could consult with a dietitian as needed, and 18 additional one-on-one sessions were conducted during the study period to evaluate and reinforce dietary adherence.

### 4.5. Statistical Analysis

To assess the normality of data distribution, the Shapiro–Wilk test was employed as an initial step. Since the majority of variables demonstrated a normal distribution, a two-way mixed-design Analysis of Variance (ANOVA) for independent samples was used to compare differences across the intervention and control groups. This analysis assessed three primary effects: group, time, and group-by-time interaction.

The group effect captures differences in outcomes across the study groups (intervention vs. control), independent of the time factor. A significant group effect suggests that the interventions produced distinct effects when compared to the control or relative to one another. The time effect reflects within-group changes over the course of the study (pre- vs. post-intervention). The interaction effect (group × time) evaluates whether changes over time differed between groups, indicating a differential impact of the interventions. Hs-CRP, IL-6 were log-transformed, with Greenhouse–Geisser correction applied when sphericity was violated; tabled values are means ± SD, while statistical testing was conducted on the transformed data.

To further explore differences between groups in response to the intervention, a two-way mixed-design ANOVA for dependent samples was conducted, followed by post hoc comparisons using Estimated Marginal Means (EMM). The magnitude of the observed effects was quantified using partial eta-squared (*η*p^2^), calculated as follows:

$${\eta p}^{2}=\frac{{SS}_{effect}}{{SS}_{effect}+{SS}_{error}}$$

The effect size was interpreted according to the following thresholds: low effect: 0.01 ≤ *η*p^2^ < 0.05, moderate effect: 0.06 ≤ *η*p^2^ < 0.13 and high effect: *η*p^2^ ≥ 0.14 [81].

In order to explain the variability in ADIPO and hs-CRP concentrations, multiple regression was applied. The models were prepared using an econometric linear model of multiple regression assessed by the method of least squares. In both models, the residual standard errors and test *p*-values were corrected using heteroscedasticity-adjusted robust standard errors.

The minimum sample size was determined using the solve_power function from the statsmodels Python library (Python Software Foundation. *Python Language and Standard Library*. Available online: https://www.python.org/ (accessed on June 2023) for repeated measures ANOVA involving a within-between interaction (two time points, three groups). Parameters included a test power of 1 − β = 0.80, a significance level of *p* = 0.05, and an effect size of d = 0.8. Under these assumptions, the required total sample size was estimated at 42 participants, corresponding to seven individuals per group.

All statistical analyses were performed according to the intention-to-treat principle, i.e., based on the participants’ originally assigned groups. Statistical significance was set at α = 0.05. All computations and visualizations were conducted using the R programming language (4.4.0) within the RStudio IDE (2024.12.1), utilizing the tidyverse, psych, corrr, emmeans, and ggplot2 packages.

## 5. Conclusions

In summary, the results of the present study demonstrate that a six-week combined resistance-aerobic training intervention paired with a high-protein, low-glycemic index diet induces more pronounced improvements in metabolic parameters, body composition, and inflammatory status than physical exercise alone. The combined intervention reduced the insulin resistance index (FG/I) and atherogenic CRI II, decreased inflammatory markers—IL-6 by 48% and hs-CRP by 30%—while simultaneously increasing ADIPO concentration by 15%. These effects were accompanied by significant changes in body composition: a reduction in visceral fat (decrease in ABD/BM) and total fat (BF/BM), an increase in fat-free mass (FFM/BM), and a decrease in the Body Adiposity Index (BAI). A crucial role in achieving these outcomes was played by dietary modification—specifically, the inclusion of low-GI carbohydrates, a 23% increase in protein intake, and a 50% increase in dietary fiber intake—which consistently deepened the energy deficit and reduced fat intake. Physical training alone also resulted in reductions in the BAI, insulin resistance (FG/I), and IL-6 levels, and initiated an increase in ADIPO secretion. These findings underscore that short-term interventions, whether training alone or combined with dietary modifications, can effectively reduce inflammation—as assessed by IL-6 and ADIPO—and lower insulin resistance in men with visceral obesity. The combined intervention leads to more favorable outcomes. Multiple regression models confirmed the cardioprotective association of ADIPO with both insulin resistance and atherogenic indices. Moreover, a correlation was established between the level of inflammation (hs-CRP) and body fat content.

Public health relevance: the intervention is pragmatic—centered on routine foods and structured, scalable training—and can be embedded in primary care and community programs. Priorities for implementation include (i) promoting concurrent training as standard of care for abdominal obesity, (ii) aligning post-exercise protein–carbohydrate intake with training days, and (iii) emphasizing low-GI, high-fiber carbohydrate sources alongside adequate protein quality. These elements can be delivered through brief counseling, digital follow-up, and partnerships with fitness facilities. Personalized treatment: given heterogeneity in adiposity distribution and metabolic status, tailoring may include adjusting resistance vs. aerobic load, setting individualized protein targets and timing, and selecting carbohydrate quality by glycemic response and tolerability. Objective monitoring (e.g., body composition, inflammatory markers) can guide iteration.

Future directions: longer, adequately powered studies should evaluate durability, adherence strategies, and hard outcomes (incident type 2 diabetes and cardiovascular events). Trials including women across hormonal stages and individuals with non-abdominal obesity are needed to test generalizability and sex-specific responses. Mechanistic studies integrating muscle/adipose biopsies and multi-omics will help link systemic biomarker changes to tissue-level pathways, clarify dose–response (training volume/intensity and protein distribution), and optimize timing/quality of post-exercise nutrition.

## Figures and Tables

**Table 1:** Daily energy expenditure associated with structured exercise (PA physical activity) and non-exercise activity thermogenesis (NEAT) in the control (CG), exercise (EG) and exercise-diet (EDG) groups.

Variable	Time	Groups Mean (SD)			Effect	*F*	*p*	*η*p^2^
		CG	EG	EDG				
Structured exercise (PA) METs	baseline week 6	0.92 (0.18) 1.00 (0.09)	1.10 (0.24) 2.97 ^a,b,c^ (0.22)	1.02 (0.04) 3.06 ^a,b,c^ (0.06)	group time interaction	432.55 1168.8 231.74	<0.01 <0.01 <0.01	0.91 0.95 0.88
NEAT METs	baseline week 6	5.01 (1.69) 5.05 (1.59)	4.50 (0.97) 4.91 (1.06)	5.53 (0.94) 5.67 (1.00)	group time interaction	2.46 1.15 0.73	0.10 0.29 0.48	0.10 0.01 0.01

METs—metabolic equivalent of task, SD—standard deviation, *p* < 0.05—statistically significant difference, *η*p^2^—effect size, ^a^ effect of group, between groups EG vs. CG: *p* < 0.01, EDG vs. CG: *p* < 0.01, after 6 weeks; ^b^ effect of time, within group *p* < 0.05 vs. baseline; ^c^ effect of intervention-time interaction *p* < 0.05 vs. baseline.

**Table 2:** Body composition: body fat to body mass (BF/BM), fat free mass to body mass (FFM/BM), abdominal fat to body mass (ABD/BM) and body adiposity index (BAI) in the control (CG), exercise (EG) and exercise-diet (EDG) groups.

Variable	Time	Groups Mean (SD)			Effect	*F*	*p*	*η*p^2^
		CG	EG	EDG				
BF/BM	baseline week 6	0.35 (0.04) 0.35 (0.04)	0.35 (0.04) 0.34 (0.04)	0.34 (0.04) 0.31 ^b,c^ (0.03)	group time interaction	2.13 21.37 11.10	0.13 <0.01 <0.01	0.09 0.03 0.03
FFM/BM	baseline week 6	0.65 (0.05) 0.64 (0.05)	0.65 (0.06) 0.66 (0.05)	0.67 (0.04) 0.69 ^b,c^ (0.04)	group time interaction	1.47 3.91 7.16	0.24 0.04 <0.01	0.06 0.01 0.02
ABD/BM	baseline week 6	0.16 (0.03) 0.16 (0.03)	0.15 (0.04) 0.15 (0.04)	0.15 (0.03) 0.12 ^b,c^ (0.03)	group time interaction	2.19 40.70 22.13	0.13 <0.01 <0.01	0.09 0.04 0.04
BAI	baseline week 6	27.65 (3.42) 27.56 (3.29)	27.48 (2.98) 26.85 ^b,c^ (2.94)	27.01 (3.28) 26.08 ^b,c^ (2.28)	group time interaction	0.43 5.73 5.40	0.65 0.02 <0.01	0.02 0.01 0.01

SD—standard deviation, *p* < 0.05—statistically significant difference, *η*p^2^—effect size, ^b^ effect of time, within group *p* < 0.05 vs. baseline; ^c^ effect of intervention-time interaction *p* < 0.05 vs. baseline.

**Table 3:** Concentrations of adiponectin (ADIPO), interleukin-6 (IL-6), high-sensitivity C-reactive protein (hs-CRP), Castelli-II Index (CRI II) and fasting glucose–insulin (FG/I) ratio in the control (CG), exercise (EG) and exercise-diet (EDG) groups.

Variable	Time	Groups Mean (SD)			Effect	*F*	*p*	*η*p^2^
		CG	EG	EDG				
ADIPO [ng/mL]	baseline week 6	4.37 (1.94) 4.49 (1.31)	3.43 (1.85) 3.97 ^b^ (1.85)	4.51 (2.03) 5.32 ^b^ (2.56)	group time interaction	1.71 6.26 0.99	0.19 0.02 0.38	0.07 0.02 0.01
IL-6 [pg/mL]	baseline week 6	12.65 (6.29) 13.34 (5.23)	8.83 (6.44) 6.17 ^a,b^ (6.17)	8.72 (5.51) 4.56 ^a,b,c^ (4.21)	group time interaction	4.86 8.67 3.86	0.01 0.01 0.03	0.18 0.03 0.03
hs-CRP [mg/L]	baseline week 6	2.43 (1.49) 2.25 (1.53)	1.95 (1.28) 1.73 (0.98)	2.69 (2.34) 1.89 ^b^ (1.55)	group time interaction	0.47 4.37 1.28	0.63 0.04 0.29	0.02 0.02 0.01
CRI II [mmol/L]	baseline week 6	2.97 (0.51) 2.77 (0.50)	2.94 (1.24) 2.73 (1.07)	2.69 (0.88) 2.47 ^b^ (0.83)	group time interaction	0.23 9.78 0.32	0.79 <0.01 0.73	0.01 0.02 0.01
FG/I	baseline week 6	7.38 (3.56) 6.75 (3.36)	6.23 (2.93) 8.57 ^b^ (3.99)	7.43 (2.65) 9.72 ^b^ (4.49)	group time interaction	0.99 5.55 2.75	0.38 0.02 0.08	0.03 0.04 0.04

SD—standard deviation, *p* < 0.05—statistically significant difference, *η*p^2^—effect size, ^a^ effect of group, between groups EG vs. CG: *p* = 0.01, EDG vs. CG: *p* = 0.01, after 6 weeks; ^b^ effect of time, within group *p* < 0.05 vs. baseline; ^c^ effect of intervention-time interaction *p* < 0.05 vs. baseline, hs-CRP, IL-6 were log-transformed, with Greenhouse–Geisser correction applied when sphericity was violated; tabled values are means ± SD, while statistical testing was conducted on the transformed data.

**Table 4:** Energy value of diet to body mass, level of proteins to body mass, carbohydrates to body mass, fiber and fats to body mass of the research participants’ diet in the control (CG), exercise (EG) and exercise-diet (EDG) groups.

Variable	Time	Groups Mean (SD)			Effect	*F*	*p*	*η*p^2^
		CG	EG	EDG				
Energy value of the diet [kcal/kg-bm]	baseline week 6	26.68 (5.13) 27.29 (4.16)	26.65 (4.33) 27.37 (4.76)	27.67 (4.47) 23.46 ^b,c^ (3.91)	group time interaction	0.56 5.62 17.36	0.58 0.02 <0.01	0.03 0.01 0.07
Proteins [g/kg-bm]	baseline week 6	1.39 (0.31) 1.33 (0.26)	1.22 (0.25) 1.20 (0.27)	1.16 (0.19) 1.50 ^b,c^ (0.27)	group time interaction	1.50 7.72 19.85	0.24 <0.01 <0.01	0.06 0.03 0.13
Carbo-hydrates [g/kg-bm]	baseline week 6	3.03 (0.76) 2.95 (0.68)	3.17 (0.85) 3.38 (0.64)	3.21 (0.71) 2.47 ^b,c^ (0.54)	group time interaction	1.61 5.19 12.86	0.21 0.03 <0.01	0.07 0.02 0.09
Fiber [g/kg-bm]	baseline week 6	0.23 (0.08) 0.23 (0.08)	0.23 (0.06) 0.23 (0.05)	0.22 (0.09) 0.33 ^b,c^ (0.09)	group time interaction	2.03 10.38 15.28	0.15 <0.01 <0.01	0.08 0.05 0.13
Fats [g/kg-bm]	baseline week 6	1.00 (0.35) 1.12 (0.27)	1.02 (0.17) 1.01 (0.27)	1.13 (0.32) 0.84 ^c^ (0.21)	group time interaction	0.32 3.21 14.19	0.73 0.08 <0.01	0.01 0.01 0.10

SD—standard deviation, *p* < 0.05—statistically significant difference, *η*p^2^—effect size, ^b^ effect of time, within group *p* < 0.05 vs. baseline; ^c^ effect of intervention-time interaction *p* < 0.05 vs. baseline.

**Table 5:** Parameters of multiple regression model of the adiponectin (ADIPO) dependent variable. Explanatory variables: Castelli-II Index (CRI II) and fasting glucose–insulin (FG/I) ratio.

Dependent Variable	Parameter Assessment	t Value	*p*-Value
Intercept	10.69 (2.61)	4.09	<0.01
CRI II	−0.72 (0.25)	−2.92	<0.01
FG/I	0.25 (0.06)	4.53	<0.01

**Table 6:** Parameters of multiple regression model of the high-sensitivity C-reactive protein (hs-CRP) dependent variable. Explanatory variables: body fat to body mass (BF/BM) and fat free mass to body mass (FFM/BM).

Dependent Variable	Parameter Assessment	t Value	*p*-Value
Intercept	−14.29 (6.43)	−2.22	0.03
BF/BM	25.50 (7.52)	3.39	<0.01
FFM/BM	11.78 (6.29)	1.87	0.07

**Table 7:** Characteristics of the research participants in the control (CG), exercise (EG), and exercise–diet (EDG) groups.

Index	Group			*p*-Value
	CG	EG	EDG	
Age [years]	34.12	34.84	34.91	0.92
	(5.52)	(6.03)	(5.61)	
FFM/BM	0.65	0.65	0.67	0.66
	(0.05)	(0.06)	(0.04)	
ABD/BM	0.16	0.15	0.15	0.69
	(0.03)	(0.04)	(0.03)	
BAI	27.65	27.48	27.01	0.69
	(3.42)	(2.98)	(3.28)	
BF/BM	0.35	0.35	0.34	0.86
	(0.04)	(0.04)	(0.04)	

CG—control group, EG—exercises group, EDG—exercises combined with diet group, fat free mass to body mass (FFM/BM), abdominal fat to body mass (ABD/BM), body adiposity index (BAI), body fat to body mass (BF/BM), *p* < 0.05—statistically significant difference.

## Data Availability

The original contributions presented in this study are included in the article and Supplementary Materials. Further inquiries can be directed to the corresponding author.

## Supplementary Materials

The following supporting information can be downloaded at: https://www.mdpi.com/article/10.3390/ijms26199500/s1.
