# Supplementary material for: Effects of Aerobic-Resistance Training and Nutritional Intervention on Adiponectin, Interleukin-6, and hs-CRP Concentrations in Men with Abdominal Obesity—A Randomized Controlled Trial
Source: Int J Mol Sci. 2025 Sep 28;26(19):9500. doi: 10.3390/ijms26199500 (PMC12525041; doi:10.3390/ijms26199500)
Supplement: Supplementary file 1 [file ijms-26-09500-s001.zip › ijms-3837217-supplementary.pdf]

Table S1. A general strategy for a combined aerobic and resistance training program intended for a groups engaged in aerobic–resistance exercises (EG and EDG).

Parameter	Warm-up (both sessions)	Session A – Push	Session B – Pull
Aerobic duration	10 min	–	–
Aerobic intensity	70 % HRmax	–	–
Strength duration	–	40 min	40 min
Strength intensity	–	70 % 1-RM	70 % 1-RM
Volume (ex. × sets × reps)	–	6 × 4 × 12	6 × 4 × 12
Rest between sets	–	1 min	1 min
Split type	–	Push	Pull
Exercises	–	1) Plank push-ups<br>2) Bench press<br>3) Standing dumbbell press<br>4) Cable press-downs<br>5) Front kettlebell squat<br>6) Plank	1) One-arm dumbbell row<br>2) Reverse-grip lat-pulldown<br>3) Bent-over dumbbell row<br>4) Dumbbell curls<br>5) Dumbbell Romanian dead-lift<br>6) Supine hip thrust
Cool-down	1 min at 50 % HRmax + stretching	–	–

(HRmax – maximum heart rate; 1-RM – one-repetition maximum).

The programme combines aerobic conditioning with resistance work in a "push–pull" split. After a 10-minute cardio warm-up performed at 70 % of maximum heart rate (HRmax), participants move to strength training, alternating two circuits:

- Circuit A ("push") – targets chest (bench press, plank push-ups), shoulders (standing dumbbell press), triceps (cable press-downs), quads (front-loaded kettlebell squat) and core (plank).
- Circuit B ("pull") – starts with three back exercises (one-arm dumbbell row, reverse-grip lat-pulldown, bent-over dumbbell row), followed by biceps curls and posterior-chain work (dumbbell Romanian dead-lift, supine hip thrust).

Each resistance block lasts 40 minutes at ~70 % 1-RM, covering 6 exercises for 4 sets × 12 reps with 1-minute rests. A brief cool-down at 50 % HRmax plus breathing and stretching for the trained muscles finishes the session.
